# Constitutive Phosphorylation of Aurora-A on Ser51 Induces Its Stabilization and Consequent Overexpression in Cancer

**DOI:** 10.1371/journal.pone.0000944

**Published:** 2007-09-26

**Authors:** Shojiro Kitajima, Yasusei Kudo, Ikuko Ogawa, Masaaki Tatsuka, Hidehiko Kawai, Michele Pagano, Takashi Takata

**Affiliations:** 1 Department of Oral and Maxillofacial Pathobiology, Division of Frontier Medical Science, Graduate School of Biomedical Sciences, Hiroshima University, Hiroshima, Japan; 2 Center of Oral Clinical Examination, Hiroshima University Hospital, Hiroshima, Japan; 3 Department of Regulatory Radiobiology, Research Institute for Radiation Biology and Medicine, Hiroshima University, Hiroshima, Japan; 4 Department of Pathology, New York University Cancer Institute, New York University School of Medicine, New York, New York, United States of America; Dresden University of Technology, Germany

## Abstract

**Background:**

The serine/threonine kinase Aurora-A (Aur-A) is a proto-oncoprotein overexpressed in a wide range of human cancers. Overexpression of Aur-A is thought to be caused by gene amplification or mRNA overexpression. However, recent evidence revealed that the discrepancies between amplification of *Aur-A* and overexpression rates of Aur-A mRNA were observed in breast cancer, gastric cancer, hepatocellular carcinoma, and ovarian cancer. We found that aggressive head and neck cancers exhibited overexpression and stabilization of Aur-A protein without gene amplification or mRNA overexpression. Here we tested the hypothesis that aberration of the protein destruction system induces accumulation and consequently overexpression of Aur-A in cancer.

**Principal Findings:**

Aur-A protein was ubiquitinylated by APC^Cdh1^ and consequently degraded when cells exited mitosis, and phosphorylation of Aur-A on Ser51 was observed during mitosis. Phosphorylation of Aur-A on Ser51 inhibited its APC^Cdh1^-mediated ubiquitylation and consequent degradation. Interestingly, constitutive phosphorylation on Ser51 was observed in head and neck cancer cells with protein overexpression and stabilization. Indeed, phosphorylation on Ser51 was observed in head and neck cancer tissues with Aur-A protein overexpression. Moreover, an Aur-A Ser51 phospho-mimetic mutant displayed stabilization of protein during cell cycle progression and enhanced ability to cell transformation.

**Conclusions/Significance:**

Broadly, this study identifies a new mode of Aur-A overexpression in cancer through phosphorylation-dependent inhibition of its proteolysis in addition to gene amplification and mRNA overexpression. We suggest that the inhibition of Aur-A phosphorylation can represent a novel way to decrease Aur-A levels in cancer therapy.

## Introduction

A series of periodic kinase reactions by cyclin-dependent kinases (CDKs) promote the progression of cell cycle [1]. Mitotic events with drastic and rapid morphological changes are also tightly regulated by other kinases including Aurora-A, -B and -C [2]. The serine/threonine kinase Aurora-A (Aur-A) is essential for mitotic entry, centrosome duplication, spindle formation, chromosome segregation and cytokinesis [3]. Human *Aur-A/STK15* is located at chromosome 20q13.2, which is commonly amplified in various cancers, including breast, colon, bladder, ovarian, pancreatic and head and neck cancers [4]–[9], and the levels of Aur-A mRNA and protein are also increased in those tumors [10]–[14]. Thus, overexpression of Aur-A kinase activity has been thought to promote carcinogenesis by disturbing the mechanism which ensures maintenance of the normal centrosome or chromosome number, perhaps due to impairment of centrosome or centromere function, cytokinesis, or spindle checkpoint regulation [2], [15], [16].

It is well established that most cell cycle regulators are degraded by the ubiquitin-proteasome system (UPS) [1], [17]. Aur-A is also degraded via the ubiquitin ligase APC (the anaphase-promoting complex) and its co-activator Cdh1 is involved [18], [19]. Proposed requirements for Aur-A ubiquitylation are recognition of the C-terminal Destruction box (D-box) by Cdh1 [20] and an additional A-box/DAD motif in *Xenopus* Aur-A [21], [22]. Furthermore, it has been suggested that Ser53 (equivalent to Ser51 in human Aur-A) of the A-box is phosphorylated during mitosis and that phosphorylation on Ser53 (or 51 in human) is essential for the mitotic stabilization of *Xenopus* [23] and human Aur-A [24]. Although the mitotic modification that affects Aur-A stabilization was discovered, the physiological dynamics and its regulation remains incompletely understood.

Previous studies have indicated that the level of Aur-A protein in tumors does not always correlate with amplification of the *Aur-A* gene [25], [26]. We also found that head and neck cancer cell lines without gene amplification expressed Aur-A protein at higher levels in comparison with those with gene amplification. In addition, a recent study using a transgenic model and derived cells has demonstrated that transgenic Aur-A protein is protected by UPS-mediated degradation during mitosis [27]. These cumulative findings led us to hypothesize that aberration of the protein destruction system induces accumulation and consequently overexpression of Aur-A in cancer. Here, we show that increased levels of Aur-A observed in head and neck cancer cell lines arise from constitutive phosphorylation of Ser51 which prevents the APC^Cdh1^-mediated ubiquitylation and sequential degradation of Aur-A.

## Results

### Overexpression of Aur-A in head and neck cancer correlates with a decrease in its degradation

**Figure 1:**
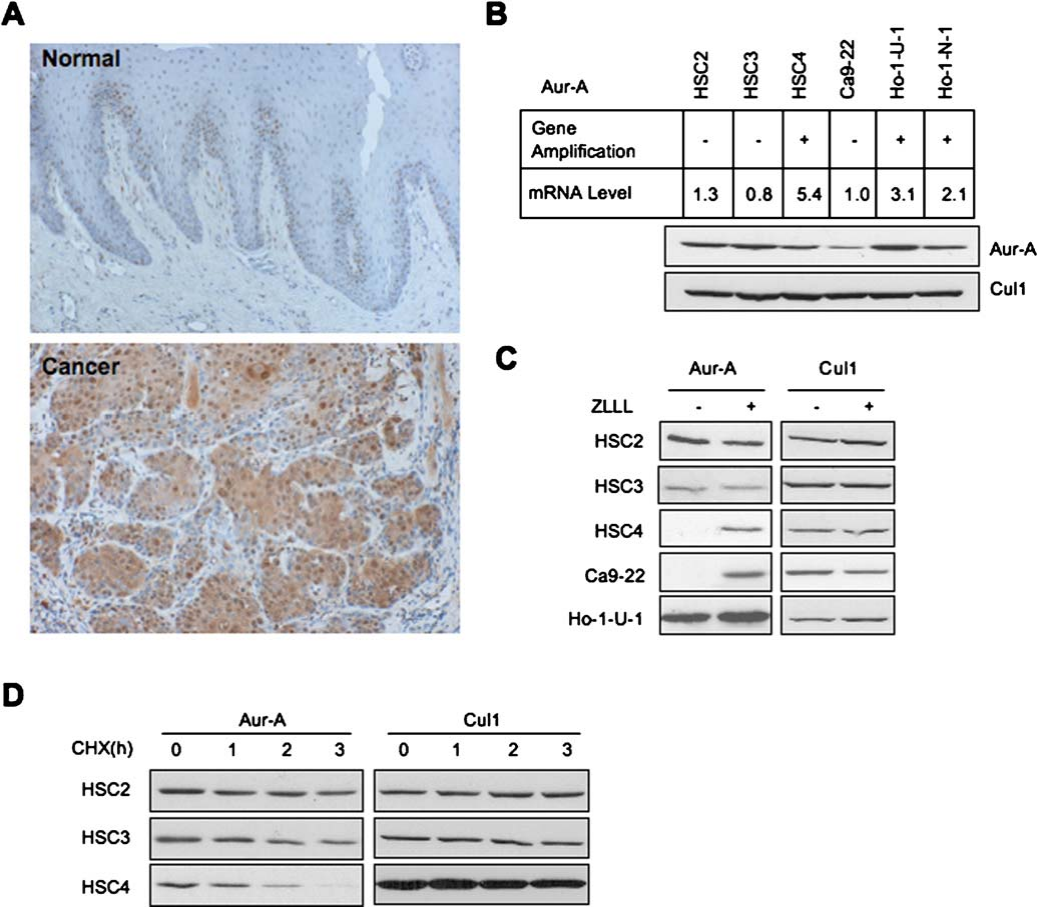
Aur-A overexpression in head and neck cancer may be caused by the abnormality of degradation. A: Immunohistochemical expression of Aur-A is shown in normal oral mucosa and head and neck cancer. B: Comparison of gene amplification, mRNA expression and protein expression in 6 head and neck cancer cell lines. Gene amplification and mRNA expression were previously examined (9). Protein expression was examined by Western blot analysis. Cul1 expression was used as a loading control. C: Accumulation of Aur-A protein by proteasome inhibitor, ZLLL. Cancer cells were treated with or without 25 µM ZLLL for 6 h. Expression of Aur-A was examined by Western blot analysis. Cul1 expression was used as a loading control. D: Half-life of Aur-A in cancer cells. Cancer cells were treated with CHX for indicated time. Expression of Aur-A was examined by Western blot analysis. Time zeros were normalized for equal amounts of Aur-A rather than equal amount of protein extracts to directly compare the two half-lives. Cul1 expression was used as a loading control.

Overexpression of Aur-A is shown in a wide range of human cancers. By immunohistochemistry, head and neck cancer cells expressed Aur-A at higher levels, in comparison with normal oral epithelial cells (Figure 1A). Importantly, Aur-A overexpression correlated with poor survival of head and neck cancer patients (supplementary table, Table S1). As Aur-A is mapped to chromosome 20q13.2, which is a region commonly amplified in epithelial malignancies, overexpression of Aur-A is thought to be caused by gene amplification and/or overexpression of mRNA. However, we found that high expression of Aur-A protein was not caused only by gene amplification and mRNA expression in head and neck cancer cell lines (Figure 1B). In particular, Aur-A protein expression in HSC2 and HSC3 cells was higher than in HSC4 cells that contain both gene amplification and elevated mRNA levels. Treatment with proteasome inhibitor, ZLLL, induced Aur-A protein accumulation in HSC4 and Ca9-22 cells, but not in those cell lines that display high expression of Aur-A (HSC2, HSC3 and Ho-1-U-1) (Figure 1C). Moreover, the half-life of Aur-A protein was longer in HSC2 and HSC3 cells than in HSC4 cells correlating with overexpressed Aur-A protein (Figure 1D). These findings led us to the hypothesis that, in addition to gene amplification or mRNA overexpression, Aur-A overexpression in head and neck cancer cells may be caused by decreased protein degradation.

### Phosphorylation of Aur-A on Ser51 inhibits APC^Cdh1^-mediated degradation

**Figure 2:**
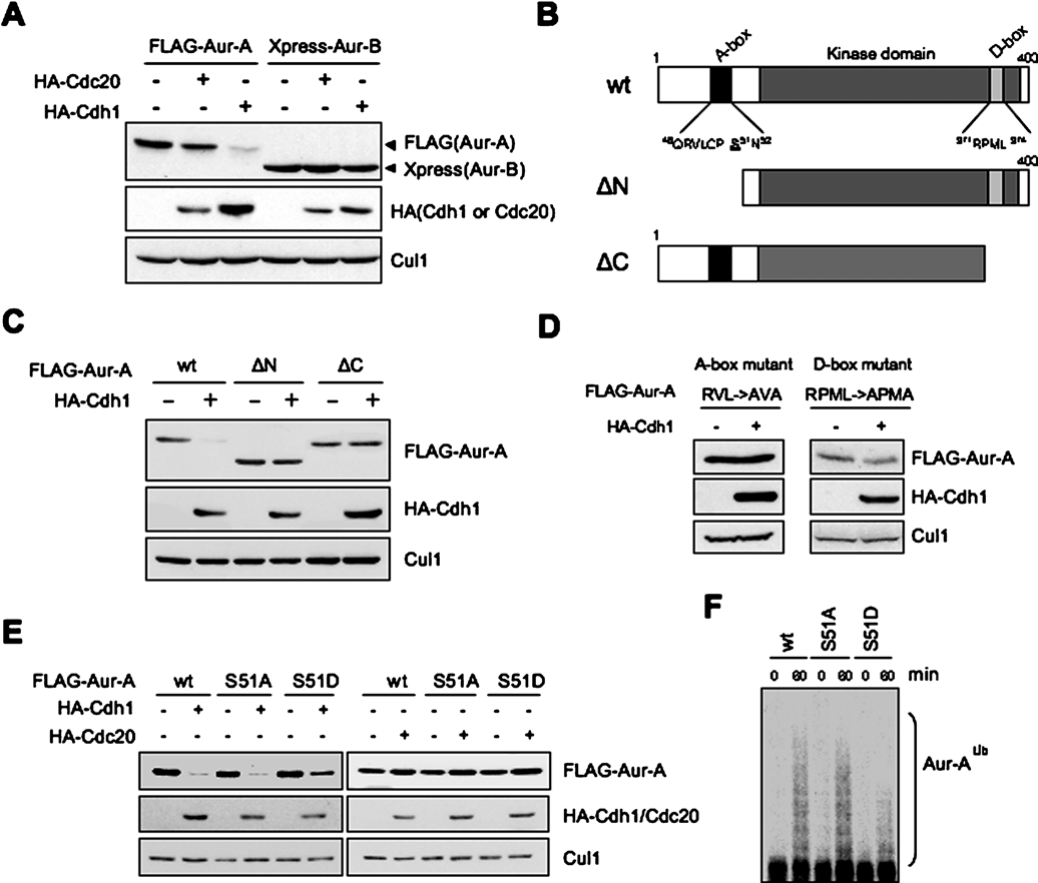
Phosphorylation on Ser51 inhibits APC^Cdh1^-mediated degradation. A: FLAG-tagged Aur-A and Xpress-tagged Aur-B were co-transfected with or without HA-tagged Cdc20 or Cdh1 in 293T cell. B: Schematic domain structure of Aur-A wild type (wt) and two deletion mutants (ΔN and ΔC) are shown. The position of two degradation motifs, A-box and D-box, are indicated. C: Aur-A-ΔN or -ΔC mutant was co-transfected with Cdh1 in 293T cell. D: A-box mutated (^46^RVL^48^ ->AVA) or D-box mutant (^371^RPML^374^ ->APMA) Aur-A was co-transfected with or without Cdh1. E: Ser51 was replaced by alanine (S51A) or aspartic acid (S51D). Each wt, S51A and S51D mutant Aur-A was co-transfected with or without Cdh1 or Cdc20. F: Sensitivity of ubiquitylation of Aur-A wt and S51 mutants were assayed *in vitro*. APC immunoprecipitated with anti-Cdc27 antibody from the HeLa cell lysates was subjected to the *in vitro* ubiquitylation assay as described in Materials and methods. The reaction was terminated at 60 min. IVT-Aur-A (arrow) was used as a substrate. “Aur-A^Ub^” indicates ubiquitylated Aur-A.

Aur-A protein expression peaks during mitosis in mammalian cells (supplementary figure, Fig. S1 A and B). ZLLL treatment induced Aur-A accumulation in cells in G1 phase, but not in cells in mitosis (supplementary figure, Fig. S1C). In fact, the protein level of Aur-A decreases in late mitosis as a consequence of ubiquitylation mediated by APC and its co-activator Cdh1 [20]. Using co-transfection experiments, we found that Aur-A protein was degraded via Cdh1, but not Cdc20 (Figure 2A), as previously reported [18], [19], [23], [24], [28]. In contrast, Aur-B, an Aur-A paralog, was not degraded by either transfection of Cdh1 or Cdc20 (Figure 2A). Next, we examined the detailed mechanism of APC^Cdh1^-mediated Aur-A degradation. Aur-A has four putative D-Box and one KEN box motifs, which could potentially be recognized by the APC^Cdh1^ ubiquitin ligase complex. In *Xenopus*, the N-terminus A-box and C-terminus D-box of Aur-A are essential for its degradation [23]. Schematic domain structure of human Aur-A wild type and two deletion mutants (ΔN and ΔC) are shown in Figure 2B. The position of two degradation motifs, A-box and D-box, are also indicated. Wild type Aur-A was degraded by co-transfection of Cdh1, while both ΔN and ΔC Aur-A mutants were not degraded (Figure 2C). The C-terminus D-box mutant and A-box mutant were also not degraded, indicating that, similarly to *Xenopus,* the A-box and D-box motifs are essential for the degradation of human Aur-A protein (Figure 2D).

It has been reported that Ser53, Thr295 and Ser349 of Aur-A are phosphorylated in *Xenopus* mitotic extracts [21]. Interestingly, phosphorylated Ser53 in *Xenopus* Aur-A blocks degradation by the UPS [23]. We generated a phosphorylation defective Aur-A mutant (Ser51 replaced by Ala; S51A) and a phospho-mimicking mutant (Ser51 replaced by Asp; S51D). Each mutant was transfected in cells with or without Cdh1 or Cdc20. Wild type and S51A mutant were almost completely degraded, when cotransfected with Cdh1, whereas the S51D mutant was degraded at a lesser extent (Figure 2E). Wild type, S51A and S51D mutants were not degraded via APC^Cdc20^ (Figure 2E). According to what found *in vivo*, the S51D mutant was less ubiquitylated *in vitro* by APC^Cdh1^ (Figure 2F). Overall, these results indicate that phosphorylation on Ser51 inhibits the D-box-dependent degradation of Aur-A occurring in G1 cells via APC^Cdh1^.

**Figure 3:**
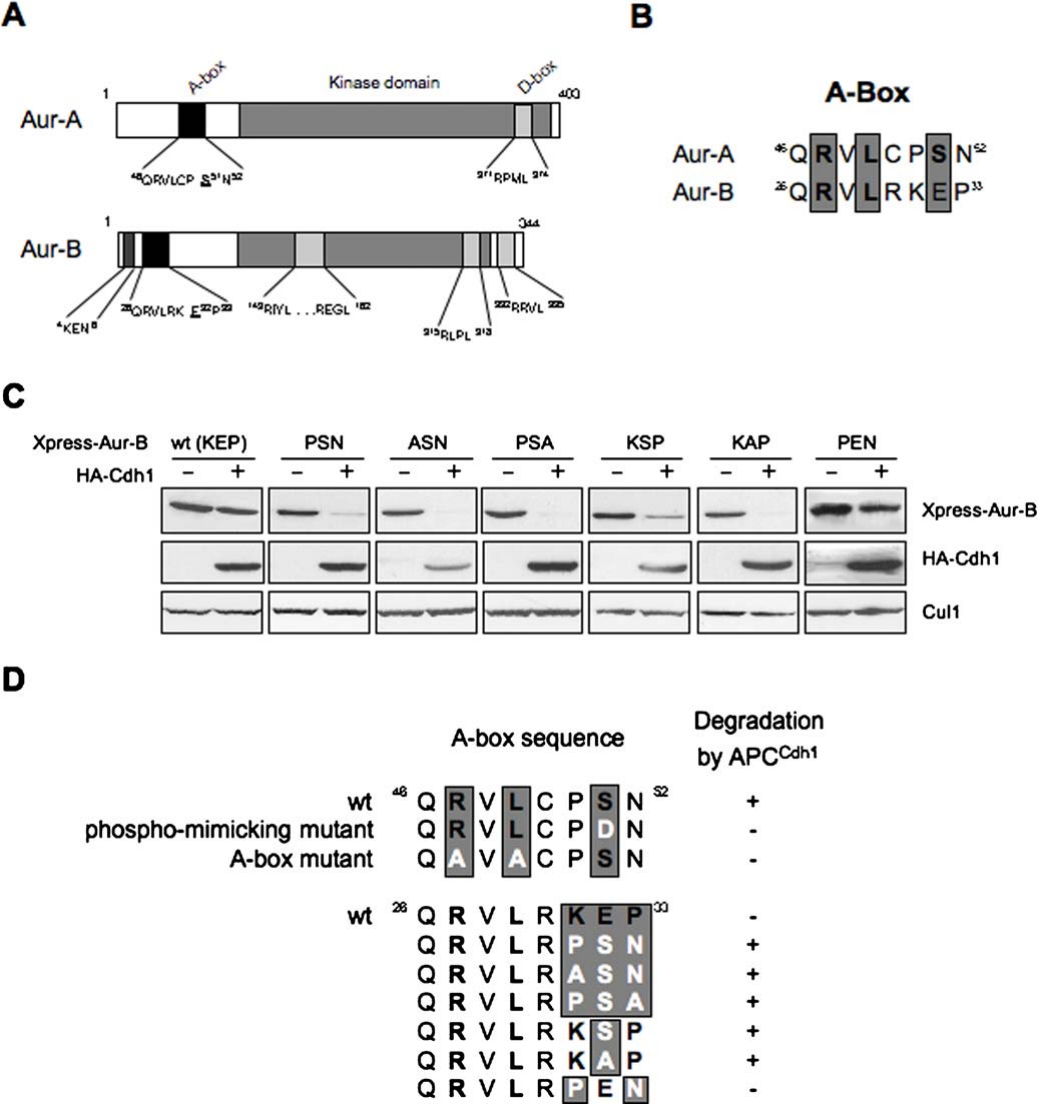
Aur-B is not degraded by APC^Cdh1^ through mimicry of phosphorylation at Glu32 in A-box. A: Comparison of schematic structure between Aur-A and -B is shown. Aur-B has several degradation motifs similarly to Aur-A. B: Corresponding amino acid sequence of A-box between Aur-A and -B is shown. C: Aur-B with mutated amino acids in A-box (^31^KEP^33^ ->PSN, ASN, PSA, KSP, KAP and PEN) was co-transfected with or without Cdh1. D: Summary of mutated sites and their results are shown.

Aurora-B (Aur-B), a paralogue of Aur-A, differs in localization and timing of activation during cell cycle from Aur-A, despite the ∼60% sequence identity between them. Comparison of the schematic structure between Aur-A and -B is shown in Figure 3A. In Similarly to Aur-A, Aur-B has one putative KEN box, four D-box and one A-box motifs. As shown in Figure 2A, however, Aur-B was not degraded by the co-expression of either Cdh1 or Cdc20. The alignment corresponding to the A-box motif of Aur-A and -B is shown in Figure 3B. Ser51 in Aur-A corresponds to Glu32 in Aur-B. We thought that Aur-B might not be degraded via APC^Cdh1^ because of Glu32 mimicking phosphorylation. To support this hypothesis, the amino acids of the Aur-B A-box were mutated (KEP ->PSN, ASN, PSA, KSP, KAP and PEN) and then transfected in cells with or without Cdh1 (Figure 3C). The schematic of the sites mutated and the results of the co-transfection experiments are shown in Figure 3D. Interestingly, PSN, ASN, PSA, KSP and KAP mutants were degraded, while PEN mutant was not degraded via APC^Cdh1^. Thus, Aur-B appears to be protected from APC^Cdh1^-mediated degradation because of Glu32 that mimics the effect of phosphorylation. All together, these results suggest that phosphorylation of Aur-A on Ser51 plays an important role for the regulation of its stability.

**Figure 4:**
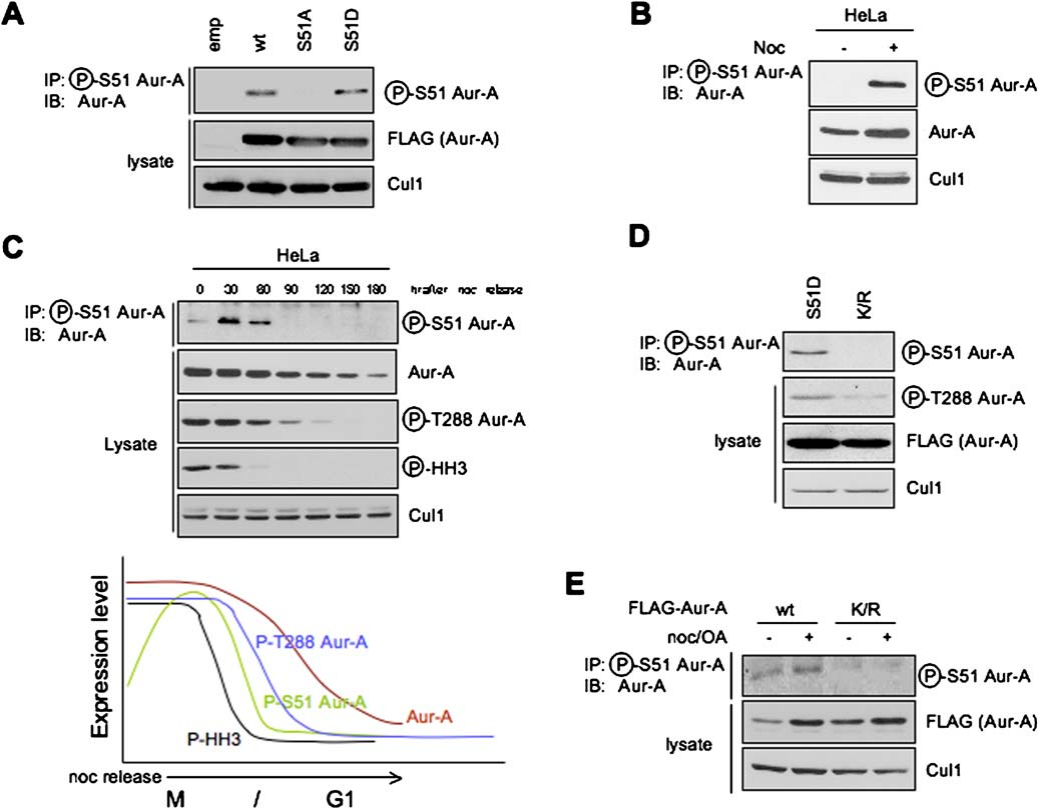
Phosphorylation on Ser51 during mitosis. A: Characterization of phosopho-specific antibody against Ser51 of Aur-A. Expression of Ser51 phosphorylated Aur-A protein is examined by immunoprecipitation (IP) with a phosopho-specific antibody against Ser51 of Aur-A followed by immunoblottoing (IB) analysis with a monoclonal antibody to Aur-A in wt and S51 mutants of Aur-A transfected 293T cells. B: Phosphorylation of Ser51 in HeLa cells with or without Noc treatment. C: Phosphorylation on Ser51 in HeLa cells. HeLa cells were released from Noc-induced prometaphase arrest and collected at the indicated times. Samples were analyzed by SDS-PAGE followed by Western blotting with phospho-S51 Aur-A, Aur-A, phospho-T288 Aur-A, phospho-histone H3 (Ser10) and Cul1 antibodies (upper panel). Graph shows expression level of Aur-A, phospho-S51 Aur-A, phospho-T288 Aur-A and phospho-histone H3 (Ser10) (lower panel). D: Expression of Ser51 phosphorylated Aur-A protein is examined by western blot analysis in S51D and K/R (kinase inactive) mutants transfected 293T cells. Phosphorylation on Thr288 was examined to demonstrate that K/R affected as a dominant negative. E: Expression of Ser51 phosphorylated Aur-A protein is examined by western blot analysis in wt and K/R mutant transfected 293T cells with nocodazole (noc) and okadaic acid (OA).

Next, we examined if phosphorylation on Ser51 was involved in regulation of Aur-A expression during cell cycle progression. We raised a phospho-specific antibody against a synthetic peptide that spans the phosphorylated Ser51 residue of Aur-A. This antibody specifically recognized wild type and S51D mutant, but not S51A mutant (Figure 4A), indicating that S51D substitution effectively mimic the negative charge of the phosphate in position 51. Phosphorylation on Ser51 in endogenous Aur-A was detected in HeLa cells treated with nocodazole (which increases the percentage of cells in mitosis), but not in those without nocodazole (Figure 4B). Ninety minutes after release from mitosis, Aur-A phosphorylated on Ser51 disappeared with decreasing protein level of Aur-A and phosphorylated Aur-A on T288 (Figure 4C). Interestingly, phosphorylation on Ser51 was not found in cells transfected with a kinase inactive mutant (K/R; K162R) (Figure 4D). In Fig. 4E, increased Ser51 phosphorylated Aur-A wt was observed after noc/OA treatment, whereas FLAG-Aur-A K/R mutant was not observed with or without noc/OA treatment. We used okadaic acid as a phosphatase inhibitor. We also used nocodazole for synchronizing the cells in mitosis when Ser51 is phosphorylated. These results indicated that the kinase activity of Aur-A is essential for phosphorylation of Ser51. The finding that Ser51 phosphorylated Aur-A was increased by noc/OA treatment is strongly supported by the recent finding that phosphorylation on Ser51 was dephosphorytated by PP2A. However, the detailed mechanism of phosphorylation on Ser51 needs further experiments.

### Aur-A overexpression in head and neck cancer cells is caused by constitutive phosphorylation on Ser51

**Figure 5:**
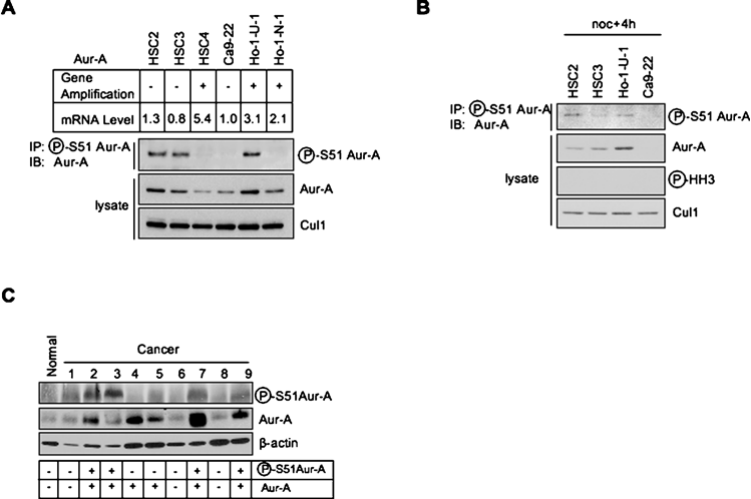
Aur-A overexpression in head and neck cancer cells is caused by phosphorylation on Ser51. A: Phosphorylation on Ser 51 in head and neck cancer cells. Expression of Ser51 phosphorylated Aur-A protein is examined by immunoprecipitation (IP) with a phosopho-specific antibody against Ser51 of Aur-A followed by immunoblottoing (IB) analysis with a monoclonal antibody to Aur-A in head and neck cancer cells. Gene amplification and mRNA expression were previously examined [9]. B: Constitutive phosphorylation on Ser 51 in head and neck cancer cells. Indicated cancer cell lines were released from noc-induced prometaphase arrest and collected in 4 h. Cells had almost completely exited from mitosis. Expression of Ser51 phosphorylated Aur-A protein is examined by immunoprecipitation (IP) with a phosopho-specific antibody against Ser51 of Aur-A followed by immunoblottoing (IB) analysis with a monoclonal antibody to Aur-A. Cul1 was used as a loading control and phospho-histone H3 (Ser10) was used as a marker for mitosis. C: Expression of Ser51 phosphorylated Aur-A protein is examined by immunoprecipitation (IP) with a phosopho-specific antibody against Ser51 of Aur-A followed by immunoblottoing (IB) analysis with a monoclonal antibody to Aur-A in cells in normal oral mucosal tissue and 9 head and neck cancer tissues. ß-Actin expession was used as a loading control.

In consideration of the above findings, we hypothesized that Aur-A overexpression in head and neck cancer cells may be caused by stabilization of Aur-A protein through a constitutive phosphorylation on Ser51. Therefore, we examined the status of phosphorylation on Ser51 in head and neck cancer cell lines. Phosphorylation on Ser51 was detected in HSC2, HSC3 and Ho-1-U-1 cells (Figure 5A). Interestingly, these cells expressed Aur-A protein at higher levels. However, HSC2 and HSC3 cells showed no gene amplification or mRNA overexpression. HSC4 cells, which display both gene amplification and high levels of mRNA, but protein levels lower than that present in HSC2 and HSC3, showed no phosphorylation on Ser51. Therefore, the status of Ser51 status appears to affect to protein expression levels. Interestingly, phosphorylation on Ser51 was also detected in HSC2, HSC3 and Ho-1-U-1 cells when the cells synchronized at G1 phase, suggesting that Ser51 was constitutively phosphorylated in cancer cells (Figure 5B). Moreover, we examined the status of phosphorylation on Ser51 in head and neck cancer cases. In fact, phosphorylation on Ser51 was detected in 4 of 9 head and neck cancer cases (Figure 5C). All cases with phosphorylation on Ser51 showed highly expression of Aur-A protein.

### Constitutive phosphorylation on Ser51 enhanced cell transformation

**Figure 6:**
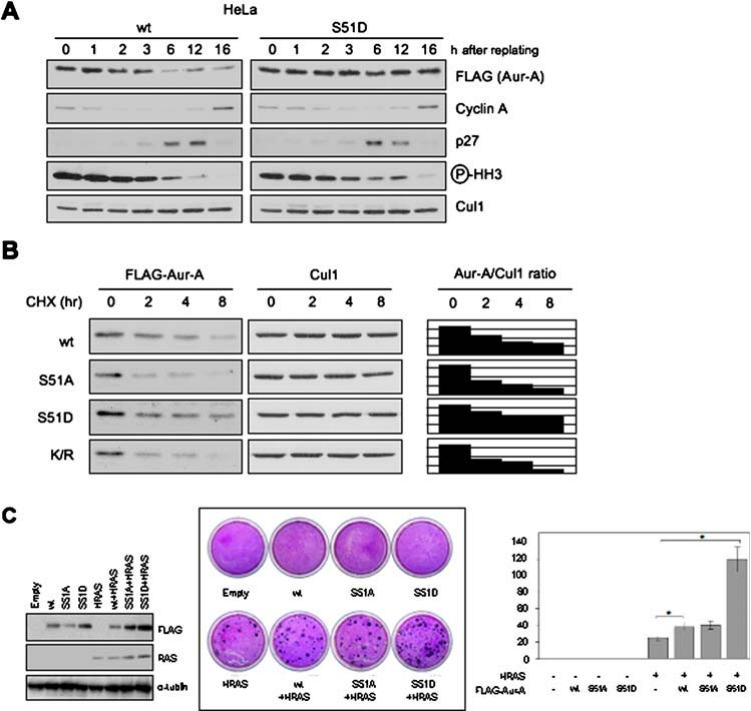
S51D mutant Aur-A enhanced cell transformation in comparison with wild type. A: Alteration of wt and S51D Aur-A expression after nocodazole release in HeLa cells. HeLa cells were transiently transfected with wt and S51D Aur-A. After 48 h of transfection, cells were synchronized by noc arrest and mitotic shake-off, released into fresh medium, harvested at the indicated times. Samples were analyzed by SDS-PAGE followed by Western blotting with FLAG, cyclin-A, p27, phospho-histone H3 (Ser10) and Cul1 antibodies. B: Half-life of wt and mutants (S51A, S51D and KR) of Aur-A transfected 293T cells (left panel). Cells were treated with CHX for indicated time. Right panel shows Aur-A/Cul1 ratio measured by densitometry. C: Effect of S51D mutant Aur-A expression on cell transformation in BALB/*c* 3T3 A31-1-1 cells. FLAG-tagged wt, S51A and S51D mutants Aur-A were transfected with or without H-Ras (G12V). Expression of wt, S51A and S51D mutants Aurora-A and H-Ras are confirmed by Western blot analysis (left panel). After 2 weeks of culture, the dishes were fixed with ethanol and stained with Giemsa solution (middle panel). Quantification of the number of transformed foci as determined using standard criteria (right panel). Error bars represent the s.d.

In order to further assess the tumorigenesis induced by overexpression of Aur-A protein due to phosphorylation on Ser51, we performed the stability of S51D mutant and cell transformation in comparison with wild type. While the expression of the wild type protein and the S51D mutant is virtually identical in mitotic cells, the S51D mutant was not degraded when cells exited mitosis (Figure 6A). In addition, the half-lives of the S51D mutant was longer than those of the wild type, S51A and K/R mutants (Figure 6B). Thus, S51D mutant was stably expressed during cell cycle progression. Then, we examined the effect of cell transformation using BALB/*c* 3T3 A31-1-1 cells (Figure 6C). We co-transfected *Aur-A* and G12V-*HRAS* (T24-*ras*), and observed that *Aur-A* potentiated the frequency of G12V-*HRAS*-induced transformation. Interestingly, a much larger number of foci were found using the S51D mutant, suggesting that constitutive phosphorylation on Ser51 has enhanced oncogenic potentials.

## Discussion

Aur-A kinase is associated with the centrosome from the time of centrosome duplication through to mitotic exit, and is also associated with regions of microtubules proximal to centrosomes in mitosis [2]. In somatic cells, both the protein levels and the kinase activity of Aur-A peak during mitosis, and then fall (supplemental figure, Fig S1) [4], [29]. It has been revealed that Aur-A is ubiquitylated by APC^Cdh1^ at the exit of mitosis [18], [19], [23], [24], [28]. The APC^Cdh1^ ubiquitin ligase complex recognizes proteins containing either D-Box or KEN-box motifs [30]–[32]. In fact, Aur-A has four D-Box and one KEN-box motifs. Here, we confirmed that the C-terminal D-box and N-terminal A-box (^47^RxLxPSN^52^) were essential for the degradation of human Aur-A, in similar to previous reports [20]–[22]. Moreover, *Xenopus* Ser53 within the A-box is phosphorylated during mitosis and that phosphorylated Ser53 (or 51 in human) is essential for mitotic specific stabilization [23], [24]. We also found that Ser51 phosphorylation inhibited APC^Cdh1^-mediated degradation. As shown in Figure 3A, Aur-B also has four D-Box, one KEN-box motifs and similar A-box sequences to Aur-A. Although it has recently been reported that protein level of Aur-B is also controlled by APC^Cdh1^ [33], [34], in our study, Aur-B expression level did not change after co-transfection with Cdh1 (Figure 2A). Interestingly, Aur-B E32A and E32S mutants (Glu32 correspond to Ser51 of Aur-A) were degraded by APC^Cdh1^ (Figure 3C and D), strongly suggesting that Aur-B may not be degraded because of phosphorylation mimicking at Glu32. Overall suggest that phosphorylation on Ser51 plays an important role for stabilization of Aur-A protein. Interestingly, phosphorylation on Ser51 was not observed in kinase inactive mutant, suggesting that Ser51 phosphorylation may be regulated at least by Thr288 phosphorylation (Figure 4D and E). Ser51 phosphorylation was observed in mitosis and disappeared before decreasing protein level of Aur-A (Figure 4C). Therefore, we suggest that Ser51 phosphorylation may control the stability of Aur-A protein level and de-phosphorylation of Ser51 may be a trigger for Aur-A degradation. Interestingly, it recently has been reported that protein phosphatase PP2A and Aur-A are co-localized at the cell poles during mitosis [35]. We found that Ser51 phosphorylation of Aur-A was induced after 2h of PP2A inhibitor treatment in HeLa cells (S. Kitajima and Y. Kudo unpublished data). These findings strongly suggest that PP2A may control Aur-A degradation by de-phosphorylating Ser51. Moreover, it is known that defects of PP2A phosphatase were detected in some cancers and several PP2A inhibitors can cause malignant alteration [36]. These findings made us hypothesize that disorder of PP2A may induce constitutive phosphorylation on Ser51 of Aur-A in cancer cells. Therefore, we examined the status of PP2A and correlated with Aur-A Ser51 phosphorylation status in head and neck cancer cell lines. However, PP2A expression was not correlated with Ser51 phosphorylation status in cancer cell lines (supplementary figure, Fig. S2). Moreover, we examined the mutation analysis of *PPP2R1B* gene, which encodes the beta isoform of the A subunit of PP2A. *PPP2R1B* was identified as a putative human tumor suppressor gene and mutation of *PPP2R1B* was observed in lung and colon cancers [37]. We could not observe any mutation of *PPP2R1B* gene in head and neck cancer cell lines (data not shown). Unfortunately, we could not find the possible correlation between PP2A and Aur-A Ser51 phosphorylation status in cancer. To demonstrate the correlation between PP2A and Aur-A Ser51 phosphorylation status needs further experiments.

Similarly to Aur-A regulation by phosphorylation, CDC6 is protected from APC-deirected degradation by virtue of it's phosphorylation [38]. Phosphorylated sites of CDC6 by cyclin E–CDK2 are located directly adjacent to the D-box, and therefore prevent recognition of CDC6 by APC^Cdh1^. In the case of Aur-A, Ser51 is located far from the D-box, but Ser51 is located in the A-box, which is also essential for ubiquitylation. However, S51D Aur-A mutant as well as wt and S51D mutant can bind to Cdh1 (supplementary figure, Fig. S3A). Surprisingly, Aur-A binds to Cdh1 and APC component, Cdc27 at M phase (supplementary figure, Fig. S3B and C). As *in vitro* ubiquitylation was inhibited in S51D mutant (Figure 2G), we suggest that Ser51 phosphorylation may disturb ubiquitylation process by APC^Cdh1^. Although the role of APC subunits in substrate recognition is more mysterious, not only the interactions between substrates and co-activators but also those between substrates and APC seem to be D-box dependent [39], [40]. Mutational analyses have shown that Doc1 is essential for the capability of APC to ubiquitylate substrates in a processive manner [41]. Therefore, phosphorylation on Ser51 may disturb the recognition by APC subunits such as Doc1, but there are a number of other possibilities. To clarify the mechanism of protection of Aur-A degradation by phosphorylation on Ser51 further studies will be required. In addition, it is interesting to examine whether or not regulation of APC mediated proteolysis by phosphorylation, as found in Aur-A and CDC6, is a common event among the other substrates.

**Figure 7:**
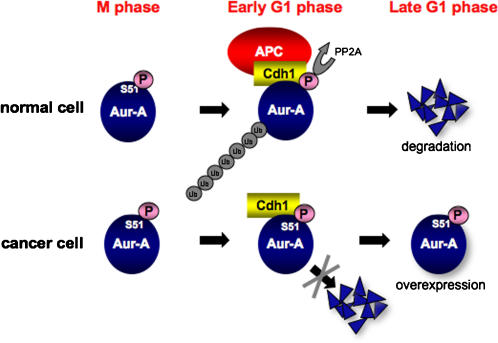
Schematic model of Aur-A overexpression in cancer. During mitosis, Aur-A is phosphorylated on Ser51 in normal cells. At mitotic exit, Aur-A is de-phosphorylated by PP2A and ubiquitylated by APC^Cdh1^. On the other hand, Aur-A is constitutively phosphorylated on Ser51 in cancer cells. Therefore, Aur-A can not be ubiquitylated and consequently accumulated in cancer cells.

Aur-A has been reported to be overexpressed in a wide range of human cancers, and its overexpression induces aneuploidy, centrosome amplification and tumorigenic transformation in cultured human and rodent cells [3]–[5]. As Aur-A is mapped to chromosome 20q13.2, a region commonly amplified in human cancers [4]–[6], overexpression of Aur-A is thought to be caused by gene amplification or transcriptional activation. In the present study, we found that high expression of Aur-A protein was not caused only by gene amplification and mRNA overexpression in head and neck cancer cell lines. This finding is supported by previous finding that amplification of *Aur-A* was detected in only 3% of cases, but more than 60% of cases overexpressed Aur-A mRNA and protein in hepatocellular carcinomas [42]. Similar discrepancies between amplification and overexpression rates were also reported in breast cancer [5], gastric cancer [26] and ovarian cancer [12]. This discrepancy may be accounted for by our findings that Ser51 constitutive phosphorylation was observed in head and neck cancer cells with overexpression of Aur-A protein. Indeed, Ser51 phosphorylation was also observed in head and neck cancer tissues with Aur-A protein overexpression. As Ser51 phosphorylation inhibited APC^Cdh1^-mediated degradation, we strongly suggest that constitutive phosphorylation on Ser51 may induce protein stabilization and consequent accumulation in cancer cells that exhibit overexpression of Aur-A protein (Figure 7). It recently has been revealed that mouse embryonic fibroblasts did not show the transformed phenotype when Aur-A was overexpressed [43], and that transgenic mice that overexpress Aur-A did not develop malignant tumors [44]. Moreover, the corresponding protein was not detected in extracts, in spite of elevated transcripts for Aur-A in multiple organs of the transgenic mice, and the treatment of transgenic-derived embryonic fibroblasts with proteasome inhibitors markedly increased the protein level of transgenic Aur-A [27]. Therefore, suppression of protein degradation might be important for Aur-A overexpression and its oncogenic role. Cell transformation by Aur-A overexpression may require suppression of protein degradation, not additional factors. Importantly, an Aur-A S51D mutant showed a significantly high susceptibility to transformation (Figure 6C). In summary, we suggest that protection of its protein degradation by constitutive phosphorylation on Ser51 may induce Aur-A overexpression in cancer, and that non-degradative Aur-A may have strong oncogenic roles. Therefore, regulation of Aur-A phosphorylation can be a novel target for cancer therapy.

## Materials and Methods

### Reagents and antibodies

Proteasome inhibitor ZLLL (Z-Leu-Leu-Leu-CHO) was obtained from Peptide institute inc. (Osaka, Japan). Cycloheximide (CHX), nocodazole (Noc) and okadaic acid (OA) were obtained from Sigma. The Aur-A phospho-Ser51-specific antibody was generated by immunizing rabbits with the synthetic peptide P*SNSSQRIPC, corresponding to amino acids 50–59 of human Aur-A sequence with a phospho-Serine at position 51 (*S). The antibody was purified from serum by two rounds of affinity chromatography on a phospho-Ser51 peptide column followed by a non-phosphopeptide column. The polyclonal antibody to cyclin A has been described previously [45]. Commercial antibodies were from the following suppliers: polyclonal antibody specific to phosphorylated Thr288 of Aur-A, Cell Signaling Technology; anti-p27 mAb, anti-Aur-A mAb and anti-Aur-B mAb, Transduction Laboratories; anti-HA polyclonal Ab (Y-11), Santa-Cruz Biotechnology; anti-phospho-histone H3 (Ser10) antibody, Upstate; anti-Cul1 polyclonal antibody, Zymed; anti-FLAG mAb (M2) and anti-RasVal12 mAb, Sigma; anti-Xpress mAb, Invitrogen; anti-α-tubulin mAb, Cedarlane Laboratories.

### Tissue samples

Tissue samples of head and neck cancer were retrieved from the Surgical Pathology Registry of Hiroshima University Hospital from 1998 to 2004 after their written informed consent. 10% buffered-formalin fixed and paraffin embedded tissues were used for immunohistochemical examination. The histological grade and stage of tumor were classified according to the criteria of the Japan Society for Head and Neck Cancer. For Western blot analysis, 9 head and neck cancer tissues and 1 normal oral mucosal tissue were obtained from patients underwent surgery at Dental hospital, Peradeniya, Sri Lanka after their written informed consent. These tissue specimens were immediately frozen and stored in −80°C. Informed consent was obtained from all patients for this study. Our work was approved by the Ethical Committee of Hiroshima University and Peradeniya University.

### Immunohistochemical staining

Immunohistochemical detection of Periostin in head and neck cancer cases was performed on 4.5 µm sections mounted on silicon-coated glass slides, using a streptavidin-biotin peroxidase technique as described previously [46]. The expression of Aur-A was graded as high (over 30% of tumor cells showed strong or diffuse immunopositivity) and low (less than 30% of tumor cells showed week, patchy or focal immunopositivity or no staining). Three pathologists (Y.K., I.O., and T.T.) made all the assessments.

### Plasmid construction and mutagenesis

Human *Aur-A* cDNA was isolated from the HeLa cDNA library by RT–PCR using sense and antisense primers. *Aur-A* cDNA was then subcloned by insertion into the *Kpn*I/*Xba*I restriction site of pcDNA3 with N-terminal FLAG tagging [9]. cDNAs were subcloned into a pcDNA3.1-His/Xpress vector (Invitrogen). cDNAs encoding His/Xpress-Aur-B was cloned into pcDNA3.1 [47]. HA-Cdc20 and HA-Cdh1 expression vectors were gift from Kristian Helin. FLAG-ΔN and -ΔC Aur-A mutants were generated by using restriction enzyme. The pcDNA3 vectors encoding the FLAG-Aur-A substitution mutants S51A, S51D, A-box mutant (RVL->AVA) and D-box mutant (RPML->APMA) and the pcDNA3.1 vectors encoding His/Xpress Aur-B substitution mutants KEP->PSN, KEP->PSA, KEP->KSP, KEP->KAP and KEP->PEN were generated with the use of a QuickChange site-directed mutagenesis kit (Stratagene).

### Cell culture

Six OSCC cell lines (HSC2, HSC3, HSC4, Ca9-22, Ho-1-U-1 and Ho-1-N-1), HeLa and 293T cells were used. All cell lines were provided by Japanese Cancer Research Resources Bank. OSCC cell lines were routinely maintained in RPMI-1640 (Kyokuto Pharmaceutical Industrial Co.) supplemented with 10% heat-inactivated fetal bovine serum (Boehringer Mannheim) and 100 U/ml penicillin-streptomycin (Gibco) under conditions of 5% CO_2_ in air at 37°C. HeLa and 293T cells were routinely maintained in Dulbecco's Modified Eagle Medium (DMEM, Nissui Pharmaceutical Co. Ldt.) supplemented with 10% heat-inactivated fetal bovine serum (Boehringer Mannheim) and 100 U/ml penicillin-streptomycin (Gibco) under conditions of 5% CO_2_ in air at 37°C. For experiments, they were grown to subconfluence in this medium.

### Transfection, immunoprecipitation, and immunoblot analysis

293T cells and HeLa were transfected with vectors with the use of FuGENE6 (Roche). Cell lysis and immunoprecipitation were performed as described [48]. Thirty μg of protein was subjected to 10% polyacrylamide gel electrophoresis followed by electroblotting onto a nitrocellulose filter. For detection of the immunocomplex, the ECL western blotting detection system (Amersham) was used. The immunoprecipitates were subjected to immunoblot analysis. For detecting phospho-Ser51 Aur-A, we performed immunoprecipitation with a phosopho-specific antibody against Ser51 of Aur-A followed by immunoblottoing analysis with a monoclonal antibody to Aur-A.

### In vitro ubiquitylation assay

[^35^S] methionine-labeled human Aur-A protein and were prepared by coupled transcription-translation reactions in rabbit reticulocyte lysate (Promega). Cold in vitro-translated human Cdh1 protein was also used. The extracts from HeLa cells were immunoprecipitated with anti-Cdc27 antibody (Sigma). Immunoprecipitants were incubated with Cdh1 in reaction mixtures contained the following in a volume of 10 µl: 40 mM Tris-HCl (pH 7.6), 1 mg/ml carboxymethyl bovine serum albumin, 1 mM DTT, 5 mM MgCl_2_, 10 mM phosphocreatine, 50 µg/ml creatine phosphokinase, 0.5 mM ATP, 50 µM ubiquitin, 1 µM ubiquitin aldehyde, 1 pmol of E1, 5 pmol of E2-C, 1 µM okadaic acid, 1-2 pmol of [^35^S] methionine-labeled human Aur-A protein. Following incubation at 30°C for 1 h, samples were subjected to electrophoresis on a 10% polyacrylamide-SDS gel.

### Transformation

The transformation target BALB/*c* 3T3 A31-1-1 cells were used in this study. For detection of oncogenes, exponentially growing cells (10^5^) were seeded in 60-mm dishes (3 dishes per experiment), after which the cells were transfected with each expression plasmid using Lipofectamine (Invitrogen). The expression plasmids for FLAG-tagged wt and mutants (S51A and S51D) were used. For the positive control, H-Ras (G12V)-induced transformation, 100 ng of mutated H-Ras plasmid (G12V; pSV2neo-ras) [49] and 900 ng empty FLAG vector [9] were mixed together and applied to each dish. After 2 weeks of culture, the dishes were fixed with ethanol and stained with Geimsa solution (Merck), observed under a dissecting microscope, and judged according to standard criteria [49].

## Supporting Information

Table S1 Summary of Aur-A expression in head and neck cancer.
(0.09 MB TIF)

Figure S1 Cell cycle-dependent regulation of Aur-A protein by APCCdh1. A: T98G cells were released from serum starvation and collected at the indicated times. Samples were analyzed by SDS-PAGE followed by Western blotting with Aur-A, Aur-B, Cyclin A, Cyclin B, p27, phospho-histone H3 (Ser10) and Cul1 antibodies. Anti-cyclin B antibody was purchased from Transduction Laboratories. B: HeLa cells were released from nocodazole-induced prometaphase arrest and collected at the indicated times. Samples were analyzed by SDS-PAGE followed by Western blotting with Aur-A, Aur-B, Cyclin A, Cyclin B, p27, phospho-histone H3 (Ser10) and Cul1 antibodies. C: Cells at M phase (noc) and G1 phase (noc+7h) were treated with or without proteasome inhibitor, ZLLL/MG132. Samples were analyzed by SDS-PAGE followed by Western blotting with Aur-A, Aur-B, Cyclin A, phospho-histone H3 (Ser10) and Cul1 antibodies.
(0.15 MB TIF)

Figure S2 Correlation between the expression of PP2A and Aur-A Ser51 phosphorylation status. Expression of PP2A (catalytic subunit alpha) is examined by Western blot analysis. We used an anti-PP2A (catalytic subunit alpha) monoclonal antibody (Transduction Laboratories). Ser51 phosphorylated Aur-A protein is examined by immunoprecipitation (IP) with a phosopho-specific antibody against Ser51 of Aur-A followed by immunoblottoing (IB) analysis with a monoclonal antibody to Aur-A in head and neck cancer cells. Gene amplification and mRNA expression were previously examined [9].
(0.08 MB TIF)

Figure S3 Aur-A binds to Cdh1 and APC component, cdc27 at M phase. A: Wild type and mutant (S51A and S51D) Aur-A bind to Cdh1. Cdh1 was co-transfected with or without Aur-A wt and two S51 mutants in 293T cell, and then ZLLL was added for 6h before the cells were collected. Cell extracts were immunoprecipitated (IP) with anti-FLAG antibody and immunoblotted with anti-Cdh1 antibody. Cul1 was used as a loading control. Aur-A wt, S51A and S51D bind to Cdh1. B: Aur-A binds to Cdh1 at M phase. After noc treatment, cell extracts were immunoprecipitated (IP) with anti-Cdh1 antibody and immunoblotted with anti-Aur-A antibody in HeLa cells. We confirmed the expression of Aur-A, Cdh1, phospho-histon H3 (P-HH3) and Cul1 in lysates. Endogenous Aur-A binds to Cdh1 at M phase. C: Aur-A binds to Cdc27 and Cdh1 at M phase. In HeLa cells at 0h and 9h after nocodazole (noc) treatment, cell extracts were immunoprecipitated (IP) with anti-Cdc27 and anti-Cdh1 antibody and immunoblotted with anti-Aur-A antibody in HeLa cells. Endogenous Aur-A binds to Cdc27 and Cdh1 at M phase. At G1 phase (noc+9h), these bindings were not observed.
(0.10 MB TIF)
