# Supplementary figures and images for: Constitutive Phosphorylation of Aurora-A on Ser51 Induces Its Stabilization and Consequent Overexpression in Cancer
Source: PLoS One. 2007 Sep 26;2(9):e944. doi: 10.1371/journal.pone.0000944 (PMC1976594; doi:10.1371/journal.pone.0000944)

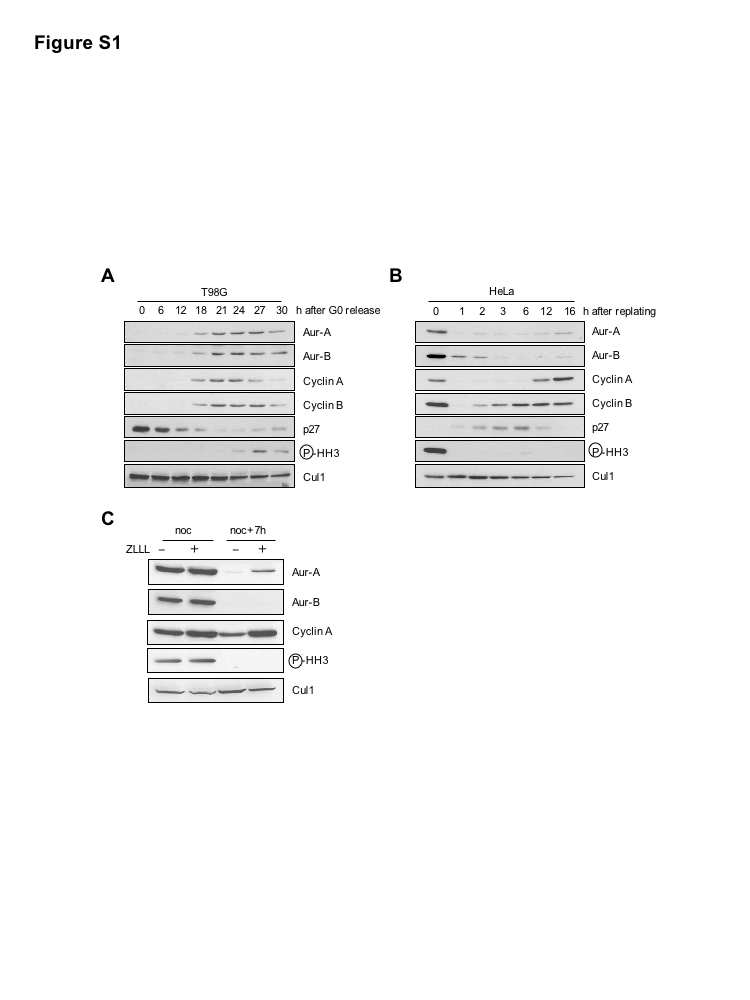

Supplement: Figure S1 — Cell cycle-dependent regulation of Aur-A protein by APCCdh1. A: T98G cells were released from serum starvation and collected at the indicated times. Samples were analyzed by SDS-PAGE followed by Western blotting with Aur-A, Aur-B, Cyclin A, Cyclin B, p27, phospho-histone H3 (Ser10) and Cul1 antibodies. Anti-cyclin B antibody was purchased from Transduction Laboratories. B: HeLa cells were released from nocodazole-induced prometaphase arrest and collected at the indicated times. Samples were analyzed by SDS-PAGE followed by Western blotting with Aur-A, Aur-B, Cyclin A, Cyclin B, p27, phospho-histone H3 (Ser10) and Cul1 antibodies. C: Cells at M phase (noc) and G1 phase (noc+7h) were treated with or without proteasome inhibitor, ZLLL/MG132. Samples were analyzed by SDS-PAGE followed by Western blotting with Aur-A, Aur-B, Cyclin A, phospho-histone H3 (Ser10) and Cul1 antibodies. (0.15 MB TIF) [file pone.0000944.s002.tif]

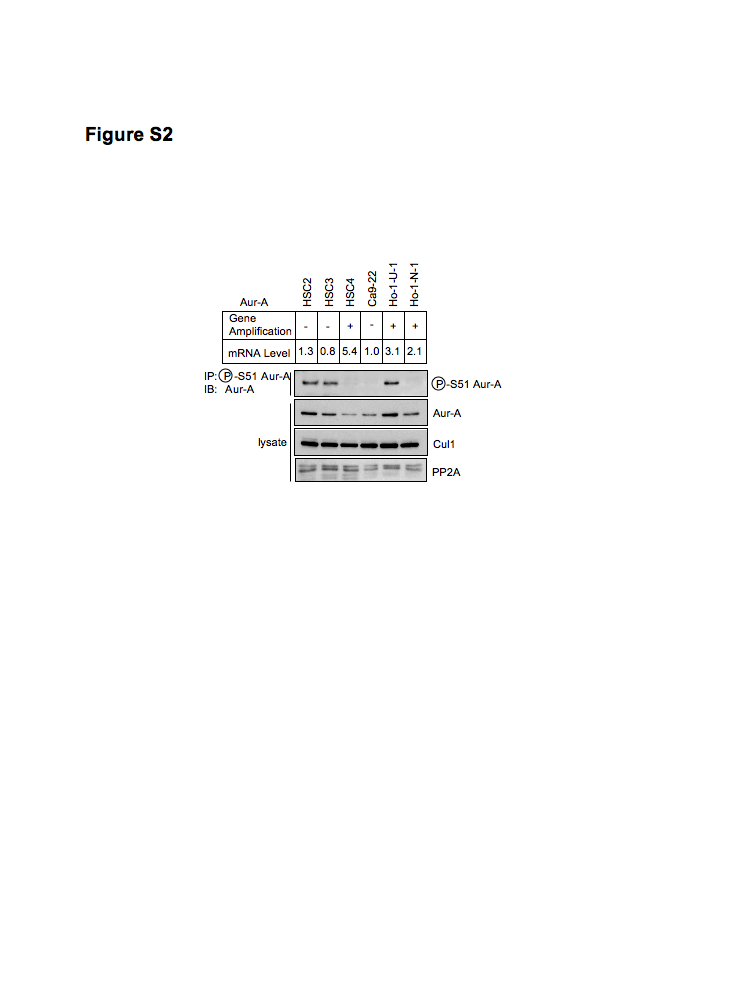

Supplement: Figure S2 — Correlation between the expression of PP2A and Aur-A Ser51 phosphorylation status. Expression of PP2A (catalytic subunit alpha) is examined by Western blot analysis. We used an anti-PP2A (catalytic subunit alpha) monoclonal antibody (Transduction Laboratories). Ser51 phosphorylated Aur-A protein is examined by immunoprecipitation (IP) with a phosopho-specific antibody against Ser51 of Aur-A followed by immunoblottoing (IB) analysis with a monoclonal antibody to Aur-A in head and neck cancer cells. Gene amplification and mRNA expression were previously examined [9]. (0.08 MB TIF) [file pone.0000944.s003.tif]

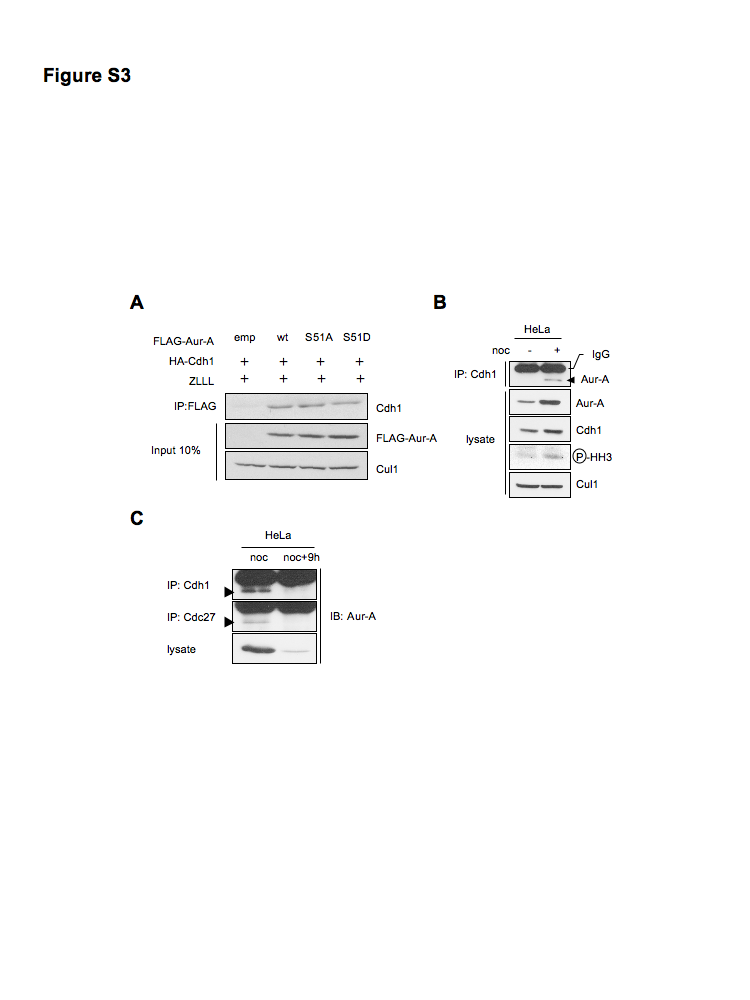

Supplement: Figure S3 — Aur-A binds to Cdh1 and APC component, cdc27 at M phase. A: Wild type and mutant (S51A and S51D) Aur-A bind to Cdh1. Cdh1 was co-transfected with or without Aur-A wt and two S51 mutants in 293T cell, and then ZLLL was added for 6h before the cells were collected. Cell extracts were immunoprecipitated (IP) with anti-FLAG antibody and immunoblotted with anti-Cdh1 antibody. Cul1 was used as a loading control. Aur-A wt, S51A and S51D bind to Cdh1. B: Aur-A binds to Cdh1 at M phase. After noc treatment, cell extracts were immunoprecipitated (IP) with anti-Cdh1 antibody and immunoblotted with anti-Aur-A antibody in HeLa cells. We confirmed the expression of Aur-A, Cdh1, phospho-histon H3 (P-HH3) and Cul1 in lysates. Endogenous Aur-A binds to Cdh1 at M phase. C: Aur-A binds to Cdc27 and Cdh1 at M phase. In HeLa cells at 0h and 9h after nocodazole (noc) treatment, cell extracts were immunoprecipitated (IP) with anti-Cdc27 and anti-Cdh1 antibody and immunoblotted with anti-Aur-A antibody in HeLa cells. Endogenous Aur-A binds to Cdc27 and Cdh1 at M phase. At G1 phase (noc+9h), these bindings were not observed. (0.10 MB TIF) [file pone.0000944.s004.tif]
